# Transthyretin Stabilization: An Emerging Strategy for the Treatment of Alzheimer’s Disease?

**DOI:** 10.3390/ijms21228672

**Published:** 2020-11-17

**Authors:** Federica Saponaro, Jin Hae Kim, Grazia Chiellini

**Affiliations:** 1 Department of Pathology, University of Pisa, 56100 Pisa, Italy; federica.saponaro@unipi.it; 2 Department of New Biology, Daegu Gyeongbuk Institute of Science & Technology (DGIST), Daegu 42988, Korea; jinhaekim@dgist.ac.kr

**Keywords:** transthyretin, protein misfolding, protein aggregation, amyloidosis, Alzheimer’s disease, TTR stabilizers

## Abstract

Transthyretin (TTR), previously named prealbumin is a plasma protein secreted mainly by the liver and choroid plexus (CP) that is a carrier for thyroid hormones (THs) and retinol (vitamin A). The structure of TTR, with four monomers rich in β-chains in a globular tetrameric protein, accounts for the predisposition of the protein to aggregate in fibrils, leading to a rare and severe disease, namely transthyretin amyloidosis (ATTR). Much effort has been made and still is required to find new therapeutic compounds that can stabilize TTR (“kinetic stabilization”) and prevent the amyloid genetic process. Moreover, TTR is an interesting therapeutic target for neurodegenerative diseases due to its recognized neuroprotective properties in the cognitive impairment context and interestingly in Alzheimer’s disease (AD). Much evidence has been collected regarding the neuroprotective effects in AD, including through in vitro and in vivo studies as well as a wide range of clinical series. Despite this supported hypothesis of neuroprotection for TTR, the mechanisms are still not completely clear. The aim of this review is to highlight the most relevant findings on the neuroprotective role of TTR, and to summarize the recent progress on the development of TTR tetramer stabilizers.

## 1. Introduction

Transthyretin (TTR) is a plasma protein secreted mainly by the liver and choroid plexus (CP) [1]. It was identified in 1942 and initially denominated “prealbumin”, as it migrates just in front of the albumin band in electrophoresis gels [2]. Later it was found that prealbumin could bind thyroid hormones (THs) [3] and retinol-binding protein (RBP) [4], therefore biochemists adopted the name “transthyretin” (TTR) to indicate its role as a transporter for thyroid hormones and retinol (vitamin A). [5]

**Figure 1:**
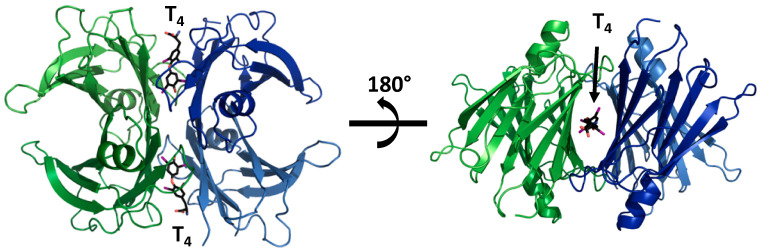
Structural model of transthyretin (TTR) complexed with T_4_ (PDB 2rox). Upon forming a tetrameric complex, TTR constitutes two hydrophobic binding pockets, which are occupied by two T_4_ molecules in this model.

Although its function may vary, TTR is a highly-conserved 55-kDa homotetrameric protein present in several vertebrate species, including humans, and also observed in bacteria, nematodes, and plants [6,7,8]. The X-ray crystal structure of human TTR shows that the protein has a globular shape, comprising four identical 127-amino-acid β-sheet-rich subunits [9]. The four monomers in the tetramer interact with each other through non-covalent bonds. A strong interaction between two monomers generates dimers that assemble as a tetramer producing a central hydrophobic channel lined by amino acids from both dimers. This channel has two similar binding sites for THs [10] (Figure 1). These binding sites can also accommodate other small compounds present in plasma, such as metabolism derivatives, components of the diet, or even small molecules administered as drugs [11]. 

**Figure 2:**
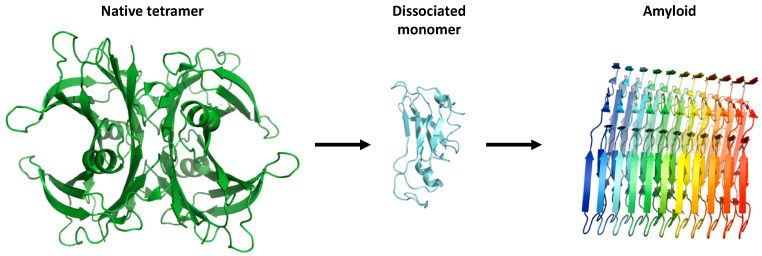
The proposed aggregation mechanism of transthyretin (TTR). The native tetrameric state of TTR [PDB 4tlt] is stable and physiologically active, while dissociation of non-native monomeric species [PDB 2nbo] initiates the pathogenic aggregation pathway of TTR. This results in accumulation of amyloid fibrils [PDB 6sdz].

The predominance of the β-chain structure in the polypeptide chains of the TTR tetramer, and its organization as β-sheets contribute to the intrinsic propensity of the protein to aggregate, leading to the formation and deposition of fibrils under specific conditions (Figure 2), thus originating transthyretin amyloidosis (ATTR), a rare, yet underdiagnosed disease characterized by progressive impairment of neurologic and cardiac function [12,13]. Notably, ATTR includes two subtypes—wild-type (ATTRwt) and variant ATTR (ATTRv)—that differ regarding the pathogenesis of amyloidosis [14]. Variant TTR deposition causes autosomal-dominant hereditary ATTR amyloidosis. The three main phenotypes of hereditary ATTR amyloidosis are familial amyloid polyneuropathy (FAP), familial amyloid cardiomyopathy (FAC), and familial leptomeningeal amyloidosis. On the other hand, wild-type ATTR deposition leads to an acquired amyloid disease, senile systemic amyloidosis (SSA), which typically develops later than hereditary ATTR [15,16]. Despite great efforts, the poor prognosis for patients with both ATTRwt and ATTRv remains unchanged, thus the availability of effective and less invasive treatments compared to liver transplantation, the current first-line treatment for hereditary ATTR amyloidosis [17], is urgently required. Over the past two decades, much has been learned about the factors that influence the propensity of TTR to aggregate [18]. Advanced biophysical information led to the development of a therapeutic strategy, termed, ‘kinetic stabilization’ to prevent wt- and v-TTR amyloidogenesis. Several stabilizing compounds that predominantly bind to the most unoccupied TH binding sites were found, the most representative being diflunisal and tafamidis [19,20].

Besides its well-known role in the transport of THs and vitamin A, TTR is increasingly recognized as possessing neuroprotective properties in multiple contexts, including cerebral ischemia and Alzheimer’s disease (AD) [21,22]. 

The earliest description of a protective role for TTR in AD dates back to 1994 when Schwarzman and co-workers reported TTR to be the major Aβ-binding protein in cerebrospinal fluid (CSF) [23]. These authors described that TTR was able to inhibit Aβ aggregation and toxicity, suggesting that when TTR fails to sequester Aβ, amyloid formation occurs [23,24]. These results have been further confirmed in various subsequent studies. In particular, studies by Buxbaum and co-workers [25] on APP transgenic mice revealed that overexpression of human (h)TTR ameliorated AD features. Increased brain Aβ levels and deposition were observed by Oliveira et al. [26] in APP transgenic mice with genetic reduction of TTR. In a triple transgenic mouse model of AD (3xTg-AD), with CP dysfunction and defective CSF production, a diminished secretion of TTR was observed [27]. Notably, Alemi et al. [28] demonstrated that TTR can transport Aβ from, but not into, the brain. Moreover, the same authors also showed that TTR increased Aβ internalization by SAHep cells (human hepatoma cells) and by primary hepatocytes derived from TTR+/+ mice when compared to TTR−/− animals [28]. Since a lower low-density lipoprotein receptor-related protein 1 (LRP1) expression was found in brains and livers of TTR−/− mice and in cells incubated without TTR, the authors proposed that TTR acts as a carrier of Aβ at the blood–brain barrier and liver, using LRP1 [28].

Despite a large body of evidence supporting the idea of neuroprotection by TTR [29,30,31,32], substantially related to decreased levels of TTR in both CSF and plasma of AD patients, the cause of TTR reduction in AD is not known yet. The aim of this review is to highlight the most relevant findings on the neuroprotective role of TTR.

## 2. TTR Physiology and Metabolism

TTR is predominantly synthesized and secreted by the liver and CP to the plasma and cerebrospinal fluid (CSF), respectively [1]. TTR plasma concentration is age-dependent, and in healthy newborns, it is about half that in adults [33,34]. TTR values vary from 20 to 40 mg/dL [33]. In spite of the low TTR levels in CSF (~2 mg/dL), the CP is presented as the major site of TTR expression, and TTR represents approximately 20% of the total CSF protein content [35]. Besides the liver and the CP, TTR is produced in the retina, pancreas (α cells), and to a small extent in the heart, skeletal muscle, stomach, and spleen [1,36,37,38]. 

TTR binds and transports about 15% of serum thyroxine (T_4_) and up to 80% of T_4_ in the CNS [39,40]. The four monomers of the TTR tetramer, demarcate through the molecule an open channel that has two binding sites for THs [41]. These two binding sites present negative cooperativity [42], implying that once the first TH molecule occupies the first site, the binding affinity for the second molecule is profoundly reduced. Thus, just one molecule of T_4_ is transported by TTR.

In addition to transporting T_4_, TTR also transports vitamin A (retinol) from its main storage site in the liver to target tissues [4]. The transport of vitamin A in circulation occurs through retinol-binding protein (RBP) [43]. TTR associates to the RBP–retinol complex before secretion into the plasma, generating a very stable form of retinol transport which allows its delivery to cells while preventing renal filtration and subsequent degradation [44,45] Under physiological conditions, due to low RBP levels compared to those of TTR, just one molecule of RBP is transported by the TTR tetramer [46,47]. Notably, T_4_ binding to TTR is not affected by RBP binding [4].

Besides its role in the transport of T_4_ and vitamin A another important function of TTR is its proteolytic activity on several substrates [48,49], including the apoliprotein A-I (apoA-I) [50], neuropeptide Y (NPY) [51], and Aβ peptide [52], further corroborating the relevance of TTR under both physiological and pathological conditions. 

## 3. The role of TTR in the Nervous System

Several reports have shown different roles for TTR in nervous system physiology [53]. Studies with TTR-null (TTR−/−) [31,54] mice revealed that these animals present reduced signs of depressive-like behavior and increased exploratory activity and anxiety, probably due to increased levels of noradrenaline in the limbic forebrain [55].

In addition, further evidence of the importance of TTR in the modulation of depressive behavior came after observing increased levels of NPY in the dorsal root ganglia (DRG), sciatic nerve, spinal cord, hippocampus, cortex, and CSF of TTR−/− mice [30,56]. Additionally, Sousa and coworkers also described that TTR−/− mice display memory impairment compared with wild-type (TTR+/+) animals, suggesting that the absence of TTR worsens cognitive deficits, a trait that is usually associated with aging [55].

In addition, studies by Fleming et al. demonstrated for the first time that TTR enhances nerve regeneration [30]. Indeed, under nerve crush conditions, the absence of TTR slowed nerve regeneration. Later, the same authors demonstrated that TTR delivery to crushed sciatic nerves rescued the regeneration phenotype in TTR-null animals, further confirming the role of TTR as a nerve regeneration enhancer [56] They also showed that the neurogenic activity of TTR is mediated by megalin-dependent internalization [56].

The neuroprotective role of TTR has been widely documented in animal models of cerebral ischemia [57,58,59]. Notably, in a mouse model of permanent middle cerebral artery occlusion (pMCAO), Santos and co-workers [29] showed that in conditions of a compromised heat-shock response, CSF TTR contributes to control neuronal cell death and cerebral edema and inflammation, thereby influencing brain protection and neuro-repair processes.

## 4. The Protective Role of TTR in Alzheimer’s Disease

Alzheimer’s disease is a neurodegenerative disorder that involves a progressive memory deficit, cognitive decline, and behavioral disturbances. The two main histopathological marks of AD are the intraneuronal presence of neurofibrillary tangles consisting of aggregates of hyper- phosphorylated tau protein, and the extracellular accumulation of senile plaques consisting of aggregates of amyloid-β peptide (Aβ) [60,61,62,63]. Lowering Aβ levels is a major therapeutic goal in AD, which might be achieved by interfering with the production, aggregation, or degradation of the peptide [64,65]

The knowledge of TTR involvement in the process of Aβ fibril formation dates back to the end of the last century [60] when it was found that TTR was able to bind Aβ40 and form stable complexes [23]. In 1986, Elovaara et al. [66] also reported the first description of decreased TTR levels in CSF of AD patients. At present it is commonly accepted by the scientific community that the neuroprotective effect of TTR in AD is linked to the following aspects: (1) TTR levels are decreased in the CSF of AD patients [67], (2) overexpressing human TTR wild type (hTTR-wt) in an AD mouse model normalizes cognition and memory [25], and (3) in vitro TTR reduces Aβ fibrillation [52,68,69]. 

TTR levels have been measured with different methods in plasma and in CSF of patients with AD and correlated with the development and prognosis of the disease. In 1997 Serot et al. obtained a total of 149 samples of CSF from control patients (*n* = 109 young, middle aged, and elderly controls) and patients with AD (*n* = 40). They measured TTR by the kinetic nephelemetric automated method and showed that TTR levels were correlated with age, but were significantly lower in patients with AD, compared with controls of the same age [67]. In 2008 Gloeckner et al. conducted a similar study on a total of 106 samples of CSF from patients with five types of dementia, that included AD and control healthy people. The levels of TTR, measured with the same nephelemetric method, were found to be significantly reduced in AD, with significantly-lower levels in patients with severe AD compared with mild forms; moreover a 15 ng/mL cut off value was established that was highly prognostic of the severity of the disease [70]. 

TTR levels were also measured in the plasma of patients with AD (*n* = 111) by ELISA (enzyme-linked immunosorbent assay) assay and they were confirmed to be significantly reduced, independently of age, when compared with controls (*n* = 90) [71]. Moreover, TTR levels have been found to correlate with AD stage and with female gender in a study by Ribeiro et al [72] and also to be predictive of cognitive decline in the ensuing months in a study by Velayudhan et al. [73] Taken together these data suggest a possible role for TTR as a peripheral biomarker for an early diagnosis of AD. 

None of the previous studies prospectively evaluated the role of TTR as biomarker of initial dementia. Very recently Tien et al. reported the results of a 5-year longitudinal study that followed the progression of 184 patients with amnestic mild cognitive impairment (MCI) and 40 sex-matched controls. In this study, TTR was demonstrated to be an independent predictor for MCI conversion to AD (*p* = 0.023, 95% CI 1–1.007) [74]. 

In animal models Choi et al. crossed mice harbouring FAD-linked APPswe and PS1_E9 transgenes with TTR knockout mice and showed that in these animals the deposition of Aβ was higher and accelerated compared with the same model and normal TTR, suggesting a role for TTR in modulating the timing and the amount of Aβ deposition [75]. These data were confirmed in the same mouse model by Oliveira et al., who also found a possible role of TTR in the modulation of the major association between female gender and AD [26].

Finally, the group of Buxbaum et al. obtained a model of APP23 AD-like and increased expression of TTR, that displayed a significant improvement in the Barnes maze test for cognitive function and spatial learning, when compared with APP23 AD-like and normal TTR expression [76].

The reason for the hypothesized TTR role in AD neuroprotection has been partially elucidated by in vitro studies. Costa et al. performed competition binding assays using soluble Aβ peptide and recombinant 125I-TTR to test the interaction between TTR and Aβ; they showed that TTR could bind Aβ, with different mutated TTRs binding Aβ with different affinities. Moreover, they found that TTR was capable of interfering with Aβ fibrillization by both inhibiting and disrupting fibril formation, since co-incubation with the two molecules resulted in the abolishment of Aβ toxicity [52]. 

Despite extensive research that spanned about three decades, the exact mechanism by which TTR modulates the process of converting monomeric Aβ into amyloid fibrils, as well as the relevance of Aβ cleavage by TTR, still remains unknown. 

Animal models of AD, such as *Caenorhabditis elegans* (CE) transgenic for human Aβ42 and TTR [77], and mice transgenic models for mutant forms of the amyloid β precursor protein (APP), expressing different levels of endogenous TTR or hTTR [78], have been widely used to dissect the mechanism behind the disease. Recently, using biophysical methods, Ghadami et al. [79] provided compelling evidence that the protective role exerted by TTR in AD, lies in the ability of TTR to inhibit the microscopic steps of both primary and secondary nucleation of Aβ aggregation, in turn limiting both the toxicity of Aβ oligomers and the ability of the fibrils to proliferate. 

In addition to TTR's well-documented ability to bind Aβ peptides, TTR proteolytic activity has been also shown to impact on Aβ fibrillogenesis [21], neuronal-secreted Aβ degradation, and reduction of Aβ-induced toxicity in hippocampal neurons [52,68], thereby contributing to the neuroprotective effect of TTR in AD. Future in vivo studies will be required to address whether TTR proteolytic activity is therapeutically relevant in AD.

## 5. Transthyretin Stability in Alzheimer’s Disease

Alterations in TTR tetramer are observed in AD. In subjects with mild cognitive impairment (MCI) reduced levels of TTR are detected, with the decrease becoming more pronounced with disease progression [80]. Although the underlying cause is not fully established, it has been proposed that TTR tetramer instability might play a role [81], suggesting that for an optimal binding to Aβ the tetramer form of TTR is required [80,82]. 

Destabilization of TTR may results from gene mutations [82]. It is widely known that TTR protein destabilized by TTR gene mutation has a tendency to dissociate into monomers, which then misfold and aggregate into amyloid fibrils, leading to autosomal-dominant hereditary amyloidosis, including familial amyloid polyneuropathy (FAP), familial amyloid cardiomyopathy (FAC), and familial leptomeningeal amyloidosis. To date, more than 140 mutations in TTR with amyloidogenic potential have been reported. The V30M TTR variant is the most common amyloidogenic form in the pathology, which leads to FAP, but other clinically-aggressive mutants have been described, including V122I, which is the most-common mutation found in cardiac amyloidosis, and L55P [26,27]. Notably, it was shown that the TTR mutation Leu-55-Pro (L55P TTR) significantly altered tetramer stability and increased amyloidogenicity under physiological conditions [72,83]. The L55P TTR tetramer was revealed to also be very sensitive to acidic conditions, readily dissociating to form the monomeric amyloidogenic intermediate between pH 5.5–5.0, while wild-type TTR remains stable and nonamyloidogenic [83].

Furthermore, T119M TTR has been described as a non-amyloidogenic transthyretin variant. Longo Alves et al. compared the stability and clearance of V30M TTR and T119M TTR and described that the more stable properties of T119M variant could be involved in the protective clinical effect of the T119M mutation in FAP. Baures et al. and Oza et al., studied several small compounds that are structurally similar to the TTR natural ligand (T_4_) and share the same TTR binding sites as T_4_, proposing them as inhibitors of TTR fibril formation in vitro [13]. In addition, with regard to the stabilization of TTR, Costa et al. described that TTR mutations, such as T119M, Y78F, V30M, and L55P, bind differently to Aβ. The observation of an inverse relationship between the amyloidogenic potential of TTR and the affinity for Aβ peptide is suggestive of a direct correlation between TTR stability and its neuroprotective properties [6].

An association of TTR variants in AD patients has been observed in a limited number of studies [84,85], therefore further studies are required to confirm whether TTR gene variability does play a major role in AD. Other events, including acidification of the medium, failure of the folding system, or interaction with various components such as metal ions and carbohydrates, may affect TTR stability [86]. Consistently, plasma TTR from AD patients showed decreased ability to bind T_4_ and decreased folded/monomeric ratios [70,71,80]. Therefore, it has been proposed that the lower concentration levels of TTR observed in the plasma of AD patients may result from the fast clearance of altered TTR. The loss in TTR tetrameric structure decreases Aβ affinity and therefore does not allow TTR to exert its protective effect in AD. Taken together this evidence has led to the hypothesis that TTR stabilization would restore both its plasma levels and proper binding to Aβ, hence its neuroprotective role in AD. 

Ultimately, it is important to mention that TTR expression, in the liver and CP, is regulated by 17β-estradiol [87]. Interestingly, it has been reported that in senescence, the AD incidence is higher in women compared to men, suggesting that estrogens may play a relevant role in this process [88]. Thus, decreased estrogens levels in AD women [80] may contribute to the decline in TTR concentration observed in AD.

In view of the fact that the amyloidogenic potential of TTR is inversely correlated with its stability, the use of drugs able to stabilize the TTR tetrameric fold could result in increased TTR/Aβ interaction. 

## 6. Transthyretin Tetramer Stabilizers

The first evidence of TTR stabilization through binding of small molecules derived from the observation that when TTR is bound to T_4_ it is less prone to aggregation. In addition, since in both the CSF and plasma the two T_4_ binding sites within TTR are largely unoccupied (<1% T_4_ bound) [9], small compounds able to bind tightly to the TTR-T_4_ binding sites may confer TTR stabilization.

Several non-steroidal anti-inflammatory drugs (NSAIDs), such as salicylates, diclofenac, flufenamic acid, and diflunisal, have been known for a long time to compete with T_4_ for the binding to TTR, [89]. Among these, diflunisal was one of the most-promising compounds due to its affinity and specificity to bind TTR. In addition, several diflunisal derivatives have been synthetized to improve its affinity and selectivity to bind TTR in plasma [90,91]. Interestingly, in a revealing experiment using an AD transgenic mouse model with TTR genetic reduction (AD/TTR+/-), administration of the iodinated derivative of diflunisal, namely iododiflunisal (IDIF) resulted in decreased amyloid burden and total Aβ brain levels, along with improved cognitive function of the animals [92]. Notably, recent data showed that the TTR/IDIF complex exhibits improved Blood Brain Barrier (BBB) permeability compared to TTR and IDIF alone [93], providing higher Aβ sequestering capacity, and adding to the therapeutic potential of TTR in AD.

Tafamidis, a benzoxazole derivative that binds to T_4_-binding sites of TTR efficiently inhibiting the dissociation of tetramers, recently emerged as a very promising drug for the treatment of familial amyloid polyneuropathy [20] and TTR-mediated amyloid cardiomyopathy (ATTR-CM) [94].

A number of therapies to reduce the amount of transthyretin protein have been in the pipeline for years. Recent studies suggested the possibility of antisense oligonucleotides (ASO) and small interfering RNA (siRNA) as genetic therapeutic agents for blocking ATTR expression. Indeed, ASOs and siRNAs could cleave mRNA prior to protein synthesis and inhibit ATTR production.

Recently, positive Phase III data on inotersen, an antisense oligonucleotide against TTR developed by Ionis Pharmaceuticals and Akcea Therapeutics, have been published [95]. Meanwhile, Alnylam has developed two RNAi drugs against TTR, namely revusiran and patisiran. These two drugs differ in their delivery strategy [96,97] —revusiran is chemically conjugated to a sugar called N-acetylgalactosamine (GalNAc), whereas patisiran is encapsulated in a nanoparticle made of a synthetic lipid called DLin-MC3-DMA [98,99] Revusiran failed in a Phase III trial [100], whereas positive Phase III results were obtained for patisiran [101]. This resulted in patisiran being the first siRNA-based drug to be approved by the U.S. Food and Drug Administration for the treatment of hereditary transthyretin amyloidosis [102,103]. Nevertheless, additional investigations on novel treatment strategies are still needed to understand the pathological role of ATTR in amyloidoses.

In the recent years, nutraceutical strategies focused on investigating the ability of natural polyphenols, such as resveratrol, to inhibit amyloid fibril aggregation, revealing for many of them neuroprotective properties in a number of experimental settings [104]. Indeed, administration of resveratrol to AD mice with TTR genetic reduction produced a significant decrease of brain Aβ accumulation and a rise in plasma TTR concentration, confirming the stabilization hypothesis. Nevertheless, different mechanisms may be involved in resveratrol action on Aβ brain levels, as it is reported that this polyphenol promotes intracellular proteasomal degradation of Aβ, while not affecting the production of the peptide [105]. Cellular studies also indicated that both resveratrol and IDIF efficiently stabilized TTR, leading to an improved TTR-assisted Aβ transport at the BBB [22].

Other polyphenols, including nordihydroguaiaretic acid (NDGA), rosmarinic acid, caffeic acid, and epigallocatechin gallate (EGCG), have also been investigated in vitro for their interaction with TTR [106,107]. The results of a small pilot clinical study with EGCG administration revealed a reduction of myocardial mass in the case of cardiomyopathy, indicating an inhibitory effect of EGCG on TTR amyloid fibril formation [108,109].

In the last fifteen years, an increasing amount of evidence underlined that in mice transgenic for human TTR V30M, supplementation with curcumin, a natural phenol produced by turmeric plants, reduces TTR load and degrades amyloid deposits in tissues, therefore targeting multiple steps in the ATTR amyloidogenic cascade [110].

In AD mice with TTR genetic reduction, evidence of a thicker basement membrane, a hallmark of AD, has been shown [51], likely reflecting vascular alterations thought to occur early, and prior to Aβ deposition, during AD development. Therefore, taking into account that currently Aβ-based therapies do not meet much favor, the stabilization of TTR can offer a new therapeutic target in the early treatment of AD.

## 7. Conclusions

Several lines of evidence suggest that TTR has a neuroprotective role in AD, and the TTR/Aβ complex is emerging as a possible new target for AD. Taking into account that, to date, there are no effective disease-slowing or -modifying treatments for AD, the discovery that TTR stabilization, through the use of small-molecule compounds, enhances the TTR/Aβ interaction, opens a new avenue that relies on the recovery of TTR activity, without affecting gene expression. Additional research will be needed to validate TTR/Aβ as a target for AD, and to move active molecules addressing this target in preclinical studies towards clinical trials for AD patients.
